# A novel blended placement model improves dietitian students’ work-readiness and wellbeing and has a positive impact on rural communities: a qualitative study

**DOI:** 10.1186/s12909-021-02756-y

**Published:** 2021-07-17

**Authors:** Narelle T Greenlees, Sabrina W. Pit, Lynda J Ross, Jo J McCormack, Lana J Mitchell, Lauren T. Williams

**Affiliations:** 1 School of Health Sciences and Social Work, Griffith University, Southport, Australia; 2 The University of Sydney, University Centre for Rural Health, Lismore, NSW Australia; 3 NSW Rural Doctors Network, Newcastle, Australia; 4 Menzies Health Institute of Queensland, Griffith University, Southport, Australia

**Keywords:** clinical training, dietetics education, practicum, placement design, rural health, work readiness

## Abstract

**Background:**

Clinical placement models that require students to relocate frequently can cause stress, which may impact the student experience and development of work-readiness skills. A blended placement, where placements are undertaken concurrently at one location has potential to address these issues by providing a positive placement experience. Blended long-stay placements undertaken in rural communities increase consistent service provision and may help encourage students to work rurally, with potential to reduce workforce shortages. The aim of this study was to pilot test the feasibility of blended placement models and explore the student experience and skills development. A secondary aim was to explore a fully blended long-stay rural placement and the benefits to the rural community.

**Methods:**

An exploratory qualitative design was used. Focus groups were conducted with dietitian student who participated in usual placements (*n* = 14) or blended placements (*n* = 9). Individual semi-structured interviews were conducted with five student supervisors who participated in blended placements. Focus groups and interviews were recorded, transcribed verbatim and analysed together using inductive thematic analyses.

**Results:**

The overarching theme across all blended model placements was ‘enhanced work-readiness’, including increased flexibility, organisational skills and better preparedness for mixed roles. Enhanced work-readiness was influenced by three themes: stress and wellbeing impacts learning, working in two areas of practice concurrently allows for deeper learning, and blended placements meet supervisor needs. Fully blended long-stay rural placements revealed additional benefits. Firstly, in relation to the overarching theme ‘enhanced work-readiness’: students on these placements also developed extra skills in innovation, social accountability, interprofessional collaboration, conflict resolution and teamwork. Secondly, a new overarching theme emerged for fully blended long-stay rural placements: ‘increased community connections’ which included additional health services delivery, deeper personal experience and more rewarding student-supervisor relationships. Thirdly, two extra themes emerged that influenced work-readiness and community impact: ‘local organisational support and resources’ and ‘enhanced innovative and interprofessional learning opportunities’.

**Conclusions:**

Blended placements enhance work-readiness skills by providing an alternative model to that commonly applied, and providing flexibility in education programs. Additionally, fully blended long-stay rural placements positively influence the local community through impacting the student experience as well as providing more dietetics services and may therefore assist in reducing dietetics workforce shortages and health inequity.

**Supplementary Information:**

The online version contains supplementary material available at 10.1186/s12909-021-02756-y.

## Background

Sub-optimal diet accounted for 11 million deaths and 255 million years of healthy life lost globally in 2017 [1]. Dietitians, the professional group qualified to treat and prevent dietary problems, therefore have a vital role in the prevention and management of chronic disease and form an integral part of the healthcare team. Systematic reviews have shown that dietetics interventions are effective in managing chronic disease [2, 3] with economic evaluation finding that dietetics intervention in primary health care leads to a significant return on investment to the health care system by keeping people out of hospital [3]. However, shortages in the dietetics workforce have been reported in the United States [4] and Canada [5], and in rural and remote areas in Australia [6]. Dietetics workforce shortages lead to reduced access to and quality of care among those who need it most. There is a clear need to resolve limited access to dietetics interventions in rural, remote and disadvantaged areas [7] to improve healthcare access and equity in rural communities.

In Australia, one-third of the population lives in a rural or remote area but is serviced by only one-quarter of the dietetics workforce [6]. Various factors contribute to this uneven distribution, one of which is a lack of familiarity with rural and remote settings [8]. There is evidence that a positive rural placement that enables students to experience rural communities and socialising leads to increased intention to work in rural or remote areas among health professional students, including dietitians [9, 10]. A qualitative national study exploring the experiences of 20 dietetics graduates [11] concluded that research ‘on role merging dietetics placements’ with support mechanisms for new dietitians is urgently needed to prepare future dietitians for practice. Universities therefore have a potential role in building the rural pipeline of the dietetics workforce.

Dietetics students in Australia undertake placement over a minimum of 100 days in three practice areas; Medical Nutrition Therapy (MNT), Food Service Management (FSM) and Community and Public Health Nutrition (CPHN). Previous accreditation standards required placements to be completed in separate blocks, and were commonly in three to four different locations, however new accreditation standards adopted in 2017 allowed for greater flexibility in placement design [12]. Blended long-stay rural placements, where placements are undertaken concurrently at one location, provide an alternative to the usual model. Rather than compartmentalise the practice areas, MNT, FSM and CPHN are interspersed, with practice areas completed part-time, at the same time, and over a longer period. The blended approach has not previously been used in Australia. However, the long-stay exposure model has been trialled and evaluated in Australia by Brown and colleagues through year-long rural dietetics placements [13]. They found that 73 % of their students undertaking a long-term placement between 2009 and 2013 gained work in a rural setting after graduation, having the potential to impact rural dietitian recruitment. A longitudinal study including these participants as well as other allied health and short-term rural placement students found 52 % of rural graduates (68 % of which were dietitians) were employed in a rural setting after one year and 37.5 % after three years [14].

Practical placements in hospitals are often synonymous with increased stress and anxiety in health professional students [15–17] which may impact the student experience and development of new graduates’ work-readiness skills. Greater expectation of skills development and increased clinical complexity in hospitals [6, 16, 18] potentially impact students in these teaching environments. Despite this increased complexity, no additional time has been mandated for dietetics placements in the last 20 years [12]. Other issues, such as financial hardship, travel and fatigue experienced on all 100-day placements can compound stress and anxiety [19]. Support networks, professional development, work role, rural lifestyle and comfort zones positively influence work-readiness among dietetic students to practice in rural locations [20]. Hence, a long-stay high-quality placement program assists work-readiness skills and helps students make informed decisions about intentions to work rurally [10].

In 2018, Griffith University, Queensland, Australia, partnered with the University Centre for Rural Health in Lismore, Northern New South Wales, Australia, to introduce a novel placement model of a blended, long-stay rural placement for a group of students from the larger dietetics cohort (the remainder of whom completed placement in the usual model). Thus students in this group undertook their entire 100 days of placement in the one rural region, in pairs. The model immersed students in real-world nutrition problems within local rural communities, allowing for deep learning of local issues across acute, primary care and preventative health, and a practical authentic learning rural experience. As well as the Northern NSW model, several Queensland sites were interested in this style of placement and undertook shorter blended placements combining MNT and FSM. The models allowed students to work in two areas of practice concurrently. It was important to evaluate how well the model worked as a teaching and learning practice, including exploring the impact of dietetics students doing long stay placements in multiple practice areas, and how this new placement model enhanced work-readiness. The Nutrition and Dietetics program at Griffith University uses design-based research to continually improve the curriculum, with students as co-designers [21]. As co-creation is an important feature of successful health intervention implementation, it is important to understand dietetics students and their supervisors’ perspectives on student work-readiness in various models of placement. This research aimed to explore perceptions of students and supervisors around student experiences and skills development during blended long-stay placement models in order to inform future practicum education. A secondary aim was to explore a fully blended long-stay rural placement and the benefits to the rural community.

## Methods

### Study design, participants and setting

Qualitative enquiry was used in the design, given the exploratory nature of the study. Focus groups were conducted with 4th -year dietetics students at the completion of their 100-day professional placement made up of MNT, FSM, and CPHN practice areas. All fully blended long-stay rural placement students were invited in person by the placement site researchers to participate in face-to-face focus groups at the placement site on the last day of placement. Purposive sampling of the non-blended cohort by a university academic occurred so as to recruit a cross-section of student experiences such as rural and urban, and a mix of successful and challenging placements. Students undertaking the non-blended model were invited via email to participate in face-to-face focus groups at the university as part of Post-Placement Week. Focus groups were conducted in June and November 2018. Supervisors who had supervised a blended placement were invited via email to participate in semi-structured individual phone interviews. Interviews were preferred over focus groups for supervisors, due to different locations and different workloads preventing a meeting at the same time. All participants were provided with a participant information sheet. Signed consent was gained prior to commencing, including permission to audio-record. The study was approved by Griffith University Human Research Ethics Committee (HREC No: GU Ref No: 2014/826).

### Data collection procedure

Each of the focus group and interview protocols were developed in consultation with clinical education staff [22], University Centre for Rural Health (UCRH) education staff and Griffith academics after a review of the literature (Appendix 1). They were not pilot tested.

#### Focus groups

Focus groups were based on the Soft Systems Methodology to analyse complexity of blended long-stay placements [23]. Specifically, the CATWOE framework was used (CATWOE = Customer, Actors, Transformation, Worldview, Ownership, Environmental constraints/aids). Focus groups were conducted by a trained dietitian/clinical educator (NG) and experienced female focus group facilitators, including a public health researcher (SWP), a public health research officer and a psychologist. The dietitian was known to the participants but not directly involved in their placements, and the group facilitators were female and unknown to the participants. Focus groups ranged between 76 and 96 min. Students interviewed underwent placement in either semester 1 or 2 of 2018 and experienced one of the following types of placement:

1. Fully blended long-stay rural placements model: This model integrated the MNT, FSM and CPHN areas of practice where students stay in the same rural region for the whole placement. Hence, this model consisted of a 5-week placement block, plus 15 weeks of blended placements, where at times the students would participate in MNT and CPHN simultaneously, and at other times in FSM and CPHN simultaneously. The students had the same primary supervisor for the duration of the placement.
2. Partially blended placements model: This model consisted of a 9-week blended MNT and FSM placement conducted in the same location, but students may have had a different supervisor and the remainder of their placement consisted of block placements in a different location.
3. Usual model: This model consisted of 2 x MNT blocks (10 weeks), a FSM block (4 weeks) and a CPHN block (6 weeks), usually at 3 or 4 locations.

#### Interviews

A dietitian/clinical educator (NG) and two FSM academics (JM, MR), all female university staff, conducted the supervisor interviews, with only JM known to the participants through prior university-placement site coordination. These interviews ranged between 18 and 32 min and were conducted between January – August 2019, reflecting their experiences of both 2018 and 2019 placements. The audio-recordings were transcribed verbatim (NG) with the help of an external transcribing service. The interviews were not reviewed by the participants.

### Data analyses

An inductive thematic analysis was conducted [24] using Word and Excel to organise the data, code and identify themes following a rigorous process outlined by Braun and Clarke [24]. NG’s student observations are included in the results and the potential bias has been acknowledged. Bias was minimised by matching the data against other supervisors and student data and continuously reviewing the findings with the research team. Transcripts were read by two researchers (NG, SWP) to identify recurrent ideas and patterns, and codes were discussed with the research team and incorporated into a codebook that continued to be refined.

Initial themes were discussed between NG, SWP and LR and further refined to enhance the quality, consistency and reliability. Themes and subthemes were organised and assigned relevant quotes from the transcripts. Direct quotes from the data were identified by focus group number and participant responses which were numbered sequentially starting at focus group 1. Themes specific to usual placements were coded, but do not form part of the discussion as the scope of the study was to explore the role of blended placements. It is unknown if data saturation was reached.

## Results

The data presented will describe the themes central to all blended placements, followed by a description of themes that relate specifically to fully blended long-stay rural placements.

### Participants

Four focus groups were conducted with 23 students (Table 1). The five supervisors were from four sites (3 rural, 1 urban) and were made up of two people who supervised both food service and MNT placements, two MNT only supervisors and one food service only supervisor.

**Table 1:** Participants

Focus group participants		
**Style of Placement**	**Gender**	**Number**
Blended	8 female, 1 male	9
Usual	13 female, 1 male	14
**Supervisors Interviewed**		
**Supervisor role**	**Location**	**Number**
MNT & FSM	Rural	2
MNT^a^ only	Urban	2
FSM^b^ only	Urban	1

^a^ MNT = Medical Nutrition Therapy; ^b^ FSM = Food Service Management

### Overview

**Fig. 1:**
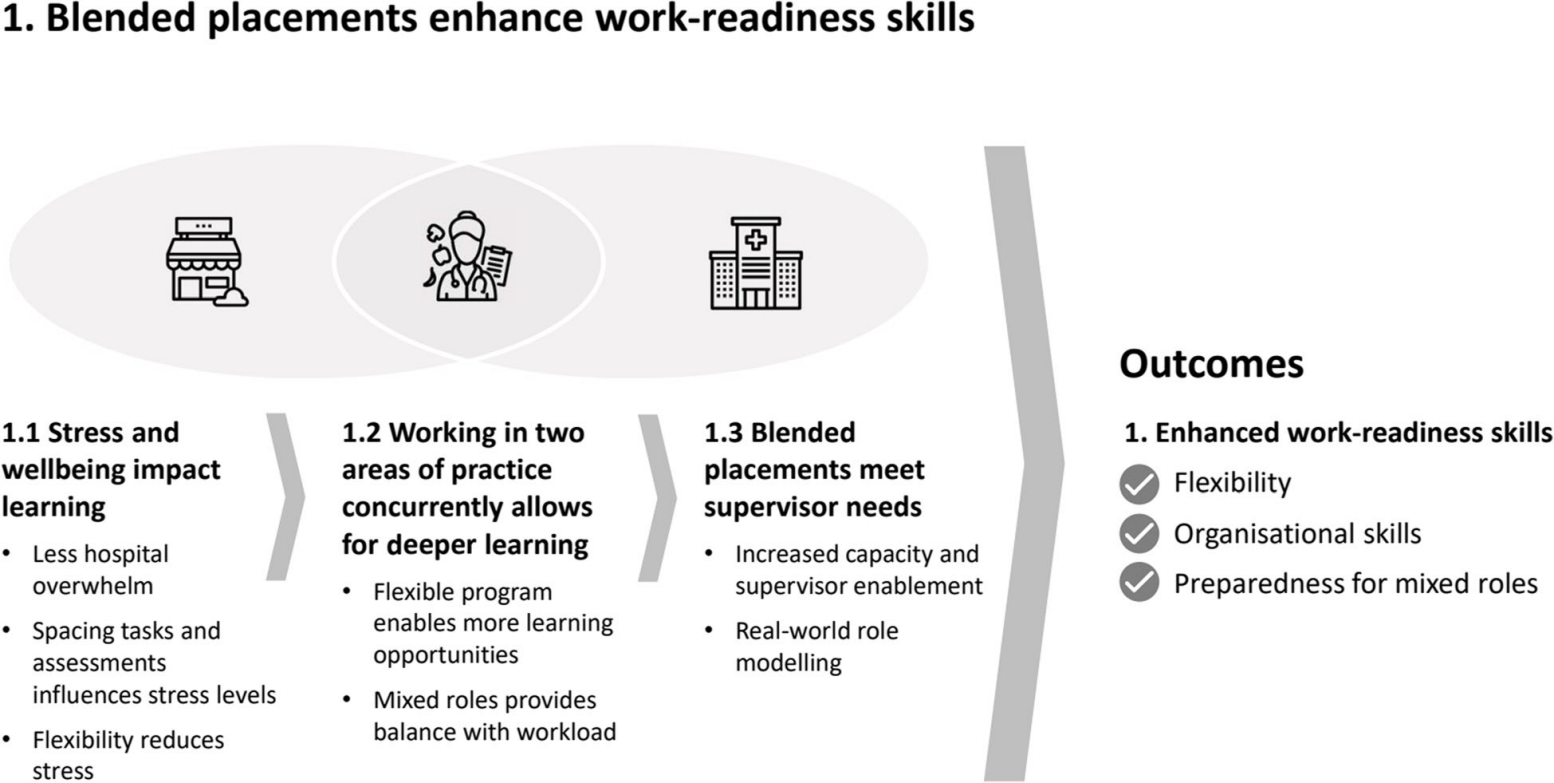
The interconnected themes about dietetic students and supervisors’ perspectives of blended placements

The overarching theme as reported by students and supervisors across all blended model placements was enhanced work-readiness, including increased flexibility, organisational skills and better preparedness for mixed roles (Fig. 1). Three sub-themes influenced blended placements leading to enhanced work-readiness: stress and wellbeing impact learning, working in two areas of practice concurrently allows for deeper learning, and blended placements meet supervisor needs.

**Fig. 2:**
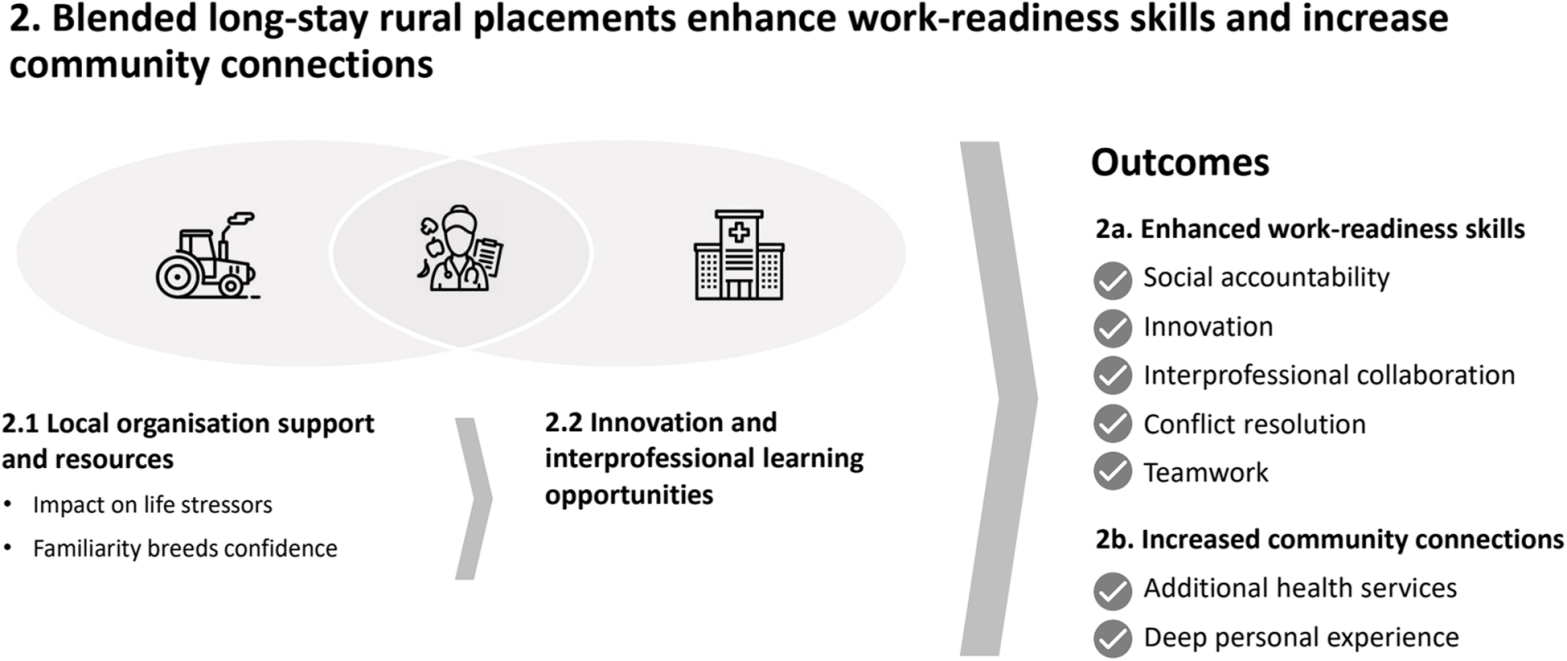
The interconnected themes about dietetic students and supervisors’ perspectives of fully blended long-stay rural placements

Fully blended long-stay rural placements revealed additional themes and sub-themes (Fig. 2). Firstly, a new theme emerged for long-stay rural blended placement: ‘increased community connections’, including ‘additional health services delivery’ and ‘deep personal experience’. Secondly, two extra sub-themes emerged that influenced ‘work-readiness’ and ‘increased community connections’: ‘local organisational support and resources’ and ‘enhanced innovative and interprofessional learning opportunities’. Thirdly, in relation to the theme ‘enhanced work-readiness’: students on these placements also developed skills in social accountability, innovation, interprofessional collaboration, conflict resolution and teamwork.

### Blended placements

#### Theme 1: Blended placements enhance work-readiness skills

Students and supervisors reported growth in students’ ability to be dynamic and flexible; increased organisational skills and being better prepared for mixed roles in the workforce.

“*When it comes to getting a job, the reality is that I might be only able to get two days working in a hospital and the other three days a week would be something else. That doesn’t seem so stressful to me anymore, because I’m used to that work balance now, and I love it. I think it’s great”* Focus group 1 #451 - blended student.

#### Sub-theme 1.1: Stress and wellbeing impact learning

Blended learning was perceived to reduce feelings of overwhelm when on hospital placements. It created a weekly balance between the highly stressful hospital clinical environment where students are under pressure to perform, constantly critiqued, and processing new information with the more independent project work in the community or food service.

*“It was challenging at times switching between the two within a week but overall I thought it was good because you got that break. Doing a three-day clinical week for me it’s quite stressful….”* Focus group 3 #556 - blended student.

Longer-stay placements also allowed students to learn more content, build confidence over a longer time period and stronger relationship with their supervisors. Students could settle in gradually, and supervisors perceived students to be less overwhelmed by the new environment than usual placement models.

*“It gives them 3 weeks settling in time where they can start to see patients, get their head around systems, as you know different hospitals have different processes, it just affords them that time”* (Supervisor 1).

Importantly, some students felt it was their responsibility to manage their stress and felt that it was easier to discuss their stress management with a supervisor with whom a trusting relationship has been developed.

*“I very openly told her when I was feeling stressed, just because – I don’t know. I suppose I just saw it as taking responsibility for myself and the way that I felt, and I didn’t enjoy those feelings. And so, I would actually let her know quite regularly how I was feeling, and it was actually in those moments where I got the most support…. Doing that actually made me feel the most supported”* Focus group 1 #142 - blended student.

While some students thrived in an environment that frequently changed during the week, some students struggled and found it challenging and a source of increased anxiety. These students had to learn to be more flexible to enable them to function in the workplace.

*“I think in the workplace now you have to be quite flexible and adaptable. And I think that this style is very good for that”* (Supervisor 2).

Students and supervisors reported some placement issues when assessments tasks were due at the same time for concurrently running placements. Whilst this contributed to students’ organisational skills development, both students and supervisors noted that subsequent placements could spread out tasks more to decrease workload stress for students.

#### Sub-theme 1.2: Working in two areas of practice concurrently allows for deeper learning

“Switching brains” was a term coined by both students and supervisors. This refers to switching thinking from a community project focus to a clinical treatment focus in the same week.

*“I certainly think they agreed with that < improved learning > in theory, but I think one of the challenges for them was changing the hat”* (Supervisor 1).

Students reported some difficulties with non-consecutive rostering for the two-site placements per week (e.g. Monday, Wednesday and Friday were hospital placements and Tuesday and Thursday were Community Project days). Supervisors also thought students would have preferred consecutive days in each setting rather than split placements throughout the week, but acknowledged this skill was important to learn for future dietetic practice.

*“Dietitians work part-time and they may work part-time as a parent or with 2 different jobs, and learning to switch your mind whilst you’re a student is an important thing to learn”* Focus Group 3 # 805 - blended student.

When complex clinical patients were admitted, students could be diverted from FSM or the community project to experience the case. One student remarked that her most rewarding clinical experience on placement was being able to follow a patient on their journey from intensive care, to the wards and then to rehabilitation over a period of 9 weeks (Focus group 3 # 603- blended student). Students reported that they could follow out-patients they had seen previously, and thereby evaluate their practice success.

*“We were able to be much more flexible in terms of the timetable to ensure that students had a much better experience and had a much wider variety in terms of Medical Nutrition Therapy exposure” (*Supervisor 2).

Supervisors were able to re-assign the student to a different task, e.g., when the next day’s outpatient appointments were cancelled, the student could be assigned to a food service day instead. Students and supervisors also reported that students could now more easily attend planned or spontaneous professional development activities.

Both supervisors and students recognised that learning was less linear on MNT placements because of the break from the clinical setting each week. Students felt this might have affected their overall competency. Comparatively, supervisors felt that overall competency was not affected and that the break from the clinical setting may have allowed for better assimilation of knowledge.

#### Sub-theme 1.3: Blended placements meet supervisor needs

The main advantage cited by supervisors was the ‘down-time’ that a blended placement affords. Clinical supervision is more resource-intensive than project supervision. The model blending these two types of work allowed supervisors to catch-up on their work whilst students worked on projects or were under another supervisor.

*“From a supervision point of view on the whole it was a much more preferred system as it enables weekly catch-up. Which means they < supervisors > can actually provide more focused supervision on the days they have the students. They can plan for it a little better. So on the whole it was definitely a plus”* (Supervisor 1).

The blended model allowed smaller rural hospitals and organisations with limited clinical caseloads to conduct blended placements, thereby increasing placement capacity in rural areas.

*“Wouldn’t be able to do a clinical block here in this rural setting as my clinical load would not be enough and productive for their learning”* (Supervisor 2).

Additionally, the rural generalist supervisors were pleased to provide real-world role-modelling of day-to-day working lives that might include inpatient and outpatient management, community liaison, education and food services management.

*“Better reflects what we do as a rural generalist”* (Supervisor 2).

### Themes unique to fully blended long-stay rural placements

#### Theme 2a: Enhanced work-readiness skills

Additional work-readiness skills, besides flexibility, organisational skills and better preparedness for mixed roles, emerged from the data related to long-stay rural placements, including the development of skills in innovation, social accountability, interprofessional collaboration, conflict resolution and teamwork. Interprofessional training, staying longer in one area, having a dedicated clinical educator and networking with different organisations and community members, allowed students to enhance their ability to innovate and increase their understanding of being socially accountable as a health professional in a rural area.

*“Because we’ve been in the community project, I feel like I’m even more aware of having different people from different walks of life, just knowing that there’s always something to learn from them, even though perhaps it’s from a completely different background. …. So, I feel like I’m a lot more open to different people’s perspectives, because there is just so much to learn from different people”* Focus group 1 #88 - blended student.

Interprofessional collaboration was increased due to exposure to other health professional groups. Conflict resolution and teamwork skills development highlighted two things; that working with the same students and supervisors over a long period can be challenging and potentially leads to conflicts; but also that a longer placement leads to improved skills in navigating these relationships and working together. Positive reflections around those challenges showed maturity and personal responsibility to make professional relationships work.

*“I think communication within the (student) partnership as well. Because even though we get along, our partnership wasn’t without issues - the only way we got past the setbacks was communicating to then move forward”* Focus group 1 #282 - blended student.

#### Theme 2b: Increased community connections

Long-stay health placements were reported by the students as having a meaningful impact on the provision of health services in rural areas and small communities and provided a deep personal experience for the students through their achievements. In terms of CPHN, students have developed and implemented programs, services, and events that may not have been possible on usual placement models.

“Community *(placement) in the normal sort of placement experience for the rest of our cohort, it’s a 6 week straight block. Whereas for us it’s been 15 weeks, so we have so much more opportunity to do more in the community. There’s no way we could have rolled out a bush tucker garden in six weeks. There’s no way. So, we were able to have such a great opportunity because it was longer time.”* Focus group 1 # 226 – blended student.

To experience firsthand the ‘lack of’ health equity in some communities and to be able to make a contribution increased their social accountability. Students felt that the size of the town had an impact on what they felt they could achieve:

*“It seems like you can make more of a meaningful impact with a community that is a small community”* Focus group 2 #432 – usual placement student.

The long-stay rural students experienced a deep personal connection through their community project work with other students, their clinical educator and supervisors, the aboriginal community and key community partners. The process was enhanced by being attached to a rural education centre, meeting friendly patients who wanted to know about them, living away from home and taking advantage of opportunities to explore the area.

*“They were very welcoming to us. We were part of the team. We felt like a part of that community and really connected, and it was really just rewarding”* Focus group 1 #212 - blended student.

Social engagement with other students and clinicians also led to deep connections in the local community and the ability to work in a team. Students gained a deeper appreciation of medicine and other allied health approaches.

*“It does add vibrance to the community. Going to cafes and seeing different students from the other unis walking around. See what they’re studying and what they’re doing is really interesting”* Focus group 3 #769 - blended student.

Students also reported feeling less intimidated by doctors and nurses because they had worked together at the education centre, increasing their sense of being part of a team.

*“The case discussions, I think for students that are intimidated with talking to nursing staff and doctors, I think it’s really important. Because I was originally quite nervous talking to them… But I think having those clinical case discussions and just being around everyone really helped and you actually felt like you were part of the team”* Focus group 1 #415 - blended student.

Supervisors felt that not only were the students more invested in the community and its relationships, but organisations and professionals were equally invested in them as students. This was felt to be extremely empowering for their sense of self-worth and their potential impact on health as future dietitians. A deep relationship with their supervisor was included in the students’ overall reflection about the connection to their placement community.

*“I think the nature of our placements in meeting all different supervisors and working with them for you know 15 weeks we have gotten to know them quite well and have developed strong networks for being from being here so long”* Focus group 3 #625 - blended student.

#### Sub-theme 2.2: Innovation and interprofessional learning opportunities

The long-stay placements enabled exposure to a range of professionals through living, clinical work and interprofessional education sessions. One group of students were able to take their food service project results and recommendations and integrate them into their clinical placement by conducting in-service sessions for nurses. The opportunity to ‘complete the circle’ of a quality improvement project was seen as very rewarding for the students. It raised their profile as professionals on the wards, empowering their role as dietitians and assisted in interprofessional skills development.

*“We did in -services to nursing staff based on results of our food service project. Based off our findings we educated nursing staff so I thought that had a good impact. The Dietetics team was so enthused that we were doing that. That we actually filled that gap. And the nurses as well gave great feedback, they all learned a lot”* Focus group 3 #821 – blended student.

Students had the opportunity to run the innovative weekly multidisciplinary Clinical Case Discussions. These are well-attended by medical practitioners, nurses and other health professionals and usually presented by medical students. Of the students who presented they later reflected that although it pushed them in terms of skills and time, they were glad they did it and learned higher order skills of facilitating a medical discussion and being organised, appreciating all roles in patient management and advocating for dietetics.

*“It made me see what a significant role we can play as dietitians, and that made me feel just really good I suppose, and I want to take that through to my own practice as well.”* - Focus Group 1 #109 - blended student.

Through weekly interprofessional learning seminars, students reported gaining a deeper appreciation of allied health approaches.

*“Well, there was different allied health professionals or students talking about their past experience and what they would do in this case, which was great, because we gained a lot of perspective of social workers and OTs”* Focus group 1 #394 - blended student.

Opportunities to learn and experience aboriginal health were also highly valued and seen as a special aspect of their placements.

*“Personally this was the best thing, the best way to learn about aboriginal health”* Focus group 3 #839 – blended student.

#### Sub-theme 2.1: Local organisation support and resources

Students recognised the benefit of a local rural educational centre providing social, economic and academic support. Students felt life stressors were reduced, particularly living arrangements, travel, finances and placement schedule changes, as they were not changing location after each placement. These students also noted growing confidence as they became familiar with their supervisors’ expectations, environment and processes.

*“One of the reasons I picked this placement, its one place. One pack. I have friends that are somewhere different for each placement. They have to organise a moving weekend, sometimes flying in between each placement. Organise accommodation on top of all the uni work, figure out where you’re going to live. Our accommodation was organised and subsidised”.* Focus group 3 #741- blended student.

The weekly interprofessional events and organised social events, were perceived as special and unique to the students on long-stay rural placements.

*“I think UCRH < University Centre for Rural Health> was great for offering those opportunities. They have emails like social connections, and events that are happening. Whereas for other placement sites it’s up to them to figure out what to do in the area. Where to go, what’s nice to see”* Focus group 3 #749 - blended student.

## Discussion

This study was the first study to qualitatively explore dietetics students and their supervisors’ perceptions of blended placement models. The students on blended placements demonstrated work-readiness skills with the blended long-stay rural placement model having additional benefits in the community. Students on blended placements demonstrated flexibility, organisational skills and preparedness for mixed roles. Students in the fully blended long-stay model also demonstrated extra skills in innovation, social accountability, interprofessional collaboration, conflict resolution and teamwork. This study has shown the impact and flexibility of different placement models.

### Blended placements

Blended placements were reported by both students and supervisors to have a positive impact on students’ stress and to provide opportunities for deep learning. Supervisors in this study felt the model met the students’ needs and resulted in ‘down-time’ from clinical learning and less hospital ‘overwhelm’. This finding adds to the limited research on stress levels among dietetics students. Indeed, Eliot and co-workers report that there is little research on stress for dietetics students on placement [25]. Eliot and colleagues [25] point to student-supervisor relationships being a source of stress due to power differences and the increased stress for supervisors in their workloads when teaching students. It appears that the blended placement model may alleviate some of these issues for both students and supervisors. Students felt it was easier to discuss stress management with their long-term supervisors, which is an important element of self-care when entering the workforce. Students described enjoying the balance of clinical versus project work in a week, which supervisors also enjoyed given it allowed them ‘catch-up’ time when the student had project work. Furthermore, blended placements included quality opportunities for learning due to timetable flexibility, and a longer placement which enabled patient follow-up over several weeks, often into the primary care and outpatient setting. The flexibility of working in two areas of practice concurrently during placements also provided learning opportunities and personal growth for the students as they needed to learn to adjust. The relevance to student training of real-world modelling of mixed task roles by supervisors observed in this study has also been demonstrated in a systematic review investigating the factors that influence dietetics students’ educational experience and work-readiness [31]. In the current study, some students were challenged by the need for flexibility and ‘switching of brains’ from patient to a project focus but this also contributed to graduate attribute skills of adaptability.

### Blended long-stay rural placements

The long-stay, blended rural placements had an additional community impact in increased health service delivery in disadvantaged areas and added personal learning experience through building strong community connections. It improved placement capacity in rural areas, which has positive effects for universities, students, the rural workforce and the community. Work-readiness was improved through enhanced innovation, social accountability, interprofessional collaboration, conflict resolution and team skills. Interprofessional collaboration will increasingly become important in the future healthcare workforce, but barriers to interprofessional collaboration in the workforce remain, such as working in silos and hierarchical structures within the healthcare system [32]. The long-stay blended placements assisted in working across different professions and learning with other health professional students. This empowered the students and increased their understanding of other professions. Practical approaches to normalising interprofessional collaboration include exposing health professional students at university to other disciplines, both socially and academically [32].

Joint Clinical Case Discussions by medical students and other allied health professions, including dietitians, were perceived as empowering by the dietetics students in our study and increased their confidence to talk to medical practitioners. Social connections with other rural health professional students and a weekly multidisciplinary academic education full day also helped to reduce interprofessional barriers through getting to know each other and increased understanding of the different roles people can play in a healthcare team. It can be argued that this may not be necessarily unique to a blended learning model, however the structure of the rural blended-model allows all different health professional students to come together on a weekly basis for a full-day over a longer period of time, which increases mutual understanding. We are unaware of this occurring elsewhere in Australia. The blended model allows students to conduct their projects over a 15 week period, compared to a non-blended placement, and to present their work back to different health professional students.

The organisational support and resources provided by the rural host-education centre for both students and supervisors were felt to influence placement success. Students had fewer life stressors on a long-stay placement such as having to relocate repeatedly, and the ability to build confidence over time because of a longer time to build relationships and recognise processes. Deep learning occurred specifically in relation to innovation opportunities in Aboriginal Torres Strait Islander people, where extended time frames enabled trusting relationships and community led programs. Similar findings were reported by Pabani and colleagues [26], who concluded that community-engaged learning environments lead to improved learning and personal and professional development. Conducting authentic, real-world projects and building connections fostered a sense of self-determination in students. Students felt rewarded and developed a sense of social accountability as a professional due to witnessing health inequity in their rural communities and providing additional health services. The findings specific to long-stay placements due to community connection is confirmed by other rural research in medicine [22], allied health [9, 10, 14] and dietetics [13]. Brown and colleagues [13] found increased employment for dietetics students in rural areas which suggests that the model can address workforce shortages in rural areas.

### Limitations

Students who self-selected blended placements may have been better prepared to do a blended model. However, all themes that emerged were consistent for both students and supervisors, increasing the strength and validity of the data. NG was one of the clinical educators and conducted most of the analyses which may have biased the results. NG applied reflexivity throughout the study through discussing her potential biases with several colleagues to minimise bias. To minimise this, she collaborated with the research members who were not involved with the program or indirectly related to the program in the analysis, interpretation, and discussion in order to form careful consensus. Furthermore, rigorous coding, e.g. through multiple iterations of refining the codebook, cross-checking and consensus discussion, was applied to ensure the richness of the data matched the themes across students and supervisors. Steps were also taken to minimise bias by including multiple experts in the interpretation, focus groups being conducted by non-clinical educators, and interview questions being developed through team collaboration. The data was collected by multiple people, potentially impacting on data collection consistency. This bias was minimised by using a structured interview protocol. Given that the blended placements were new, the student supervisors had limited experience with blended placements from a small number of students and locations at one university.

### Implications and future research

Specific recommendations for program improvement are provided in Table 2. As the program evolves, it will continuously be evaluated and improved. Additional evaluation with a larger sample size would increase the validity of these findings and help to improve the model further. An end of placement online survey would be the most efficient means of collecting feedback for both students and supervisors as well as utilising a validated Student Satisfaction Survey for Clinical Education Placements [29] or allied health specific Placement Quality Surveys that have been tested in both rural and urban areas [30]. This study has shown that a flexible approach to placement models, supported by newer and more adaptive accreditation standards [12], has benefited students learning. Lastly, we did not compare rural blended models with usual rural placement models. We acknowledge that there are usual rural placement models within Australia which may have achieved similar outcomes. This is an area for future research. Comparing blended long-stay models with usual long-stay models is also worthy of further investigation.

**Table 2:** Recommendations to improve blended dietetics placements

Preparation
• Training students on the blended model (background, evaluation results, gains for the student, and listening to past students about what to expect) could assist in their preparation for this placement model.• Supervisors require support and training to develop and undertake a differing model of placement. A site supervisor training package should include timetabling, monitoring student progression, communication and potential risks.• Students could preference placements based on the model being offered.
Placement features
• Blending clinical and food services projects appears to be the preferred model.• Schedule practice areas on consecutive days during the week.• Ensure a balanced workload for students by spreading out due dates for assessments, tasks and reports.
Supervisors
• Provide a consistent supervisor over a longer period as this enhances learning and relationships.
Administration
• University employed or funded clinical educators support placements.• Affordable and comfortable accommodation for more extended stay placements.
Community
• Create community-based projects to increase innovation, social accountability, and enhance rural health service delivery.• Interprofessional social and other learning opportunities contribute to work-readiness skills such as teamwork and interprofessional collaboration.

This study showed that blended placements encourage workforce-readiness skills and rural community connections among dietetics students. This was enabled by local organisational support and resources. This in turn may influence students’ intention to work in a rural area [9, 10]. To encourage students to feel confident to practice rurally after graduation, universities can team up with rural workforce organisations to demonstrate that rural organisational support and resources are available after graduation. The Rural Doctors Network’s Rural Health Pro [27] may help address some of the recommendations for facilitating dietitians working in rural areas. Rural Health Pro was developed to connect people and organisations who wish to keep rural communities healthy and provide a virtual networking forum for rural health professionals to combat professional isolation, and provide training, mentoring and support for rural clinicians and their family. [27]. As pointed out by Morgan and colleagues [28], greater workforce engagement is needed to benefit the dietetic profession. Virtual platforms such as Rural Health Pro may assist in this area.

## Conclusions

Blended placements provide students with work-readiness skills, and blended long-stay rural placements have the advantage of impacting on the community through additional health service delivery and deep personal experiences. Recommendations are required to ensure that students and their supervisors can function optimally during blended placements. Lessons learned and themes that emerged from the current study will assist in planning for the implementation of blended placement models and more flexible models. Embedding blended placements into the curriculum can help prepare the future dietetics workforce to ensure deep learning, community connections and potentially improve healthcare access for rural Australians.

## Supplementary information

**Additional file 1**

## Data Availability

The datasets generated and/or analysed during the current study are not publicly available due to ethics limitations but are available from the corresponding author on reasonable request.

## Abbreviations

FSM: Food Services Management
MNT: Medical Nutrition Therapy
CPHN: Community and Public Health Nutrition
