# Supplementary material for: A novel blended placement model improves dietitian students’ work-readiness and wellbeing and has a positive impact on rural communities: a qualitative study
Source: BMC Med Educ. 2021 Jul 17;21:387. doi: 10.1186/s12909-021-02756-y (PMC8286607; doi:10.1186/s12909-021-02756-y)
Supplement: Supplementary file 1 — Additional file 1 [file 12909_2021_2756_MOESM1_ESM.docx]

# **Focus Groups for students**

Introduce the following questions in their category to provide context for the participants

| **DOMAIN** | **Questions** | **Prompts applied to all placements models** |
| --- | --- | --- |
| **Preparation** | 1. You all participated in Preparation for Placement Week at University prior to commencing your placements. In particularly, you participated in the one-day ward simulation. Can you describe how the simulation prepared you, or didn’t prepare you for your placement? | - Facilitator FYI: The ward simulation took place on Day 3 of Preparation for Placement Week. Its focus was on reducing nerves and working w peers on the PAL model used in ICM |
| **Overall Experience** | 1. You have had one, or more than one supervisor whilst on placement. Can you describe how this supervision influenced your learning? | - Reaction to supervision model *(Kirkpatrick Leaning level1)* - Influence on knowledge/ skill/ attitudes *(Kirkpatrick Leaning level2)* - Ability to apply learnings in the workplace/ real world solutions (behaviour) *(Kirkpatrick Leaning level 3)* - Has the training changed processes or outcomes (eg improved quality/ efficiency), *(Kirkpatrick Leaning level 4)* |
|  | 1. Do you feel your placement model influenced your ability to feel part of the community and get to know local issues/systems? | - Individual patients - Organisation - Wider community - Wider systems - Please provide examples |
| **Day to Day Management** | 1. You may have been paired with a number of different students whilst on placement. Can you describe your experiences of these different student partnerships/ relationships (and how this influenced your learning)? | - Reaction to student partnership *(Kirkpatrick Leaning level1)* - Influence on knowledge/ skill/ attitudes *(Kirkpatrick Leaning level2)* - Ability to apply learnings in the workplace/ real world solutions (behaviour) *(Kirkpatrick Leaning level 3)* - Has the training changed processes or outcomes (eg improved quality/ efficiency), *(Kirkpatrick Leaning level 4)* |
|  | 1. Can you describe your experience of transitioning from Clinical 1 (ICM1) to Clinical 2 (ICM2) whilst on placement? | - Different site/systems - Different supervisors - Differing expectations - Differing caseload |
|  | 1. Were there particular times that were more stressful or anxiety provoking than others? Describe the impact of your placement model on this? |  |

**Note: Questions below have been based on the Soft Systems Methodology to analyse complexity and develop and refine programme theory (Dalkin, 2017). The CATWOE framework has been used (CATWOE=Customer, Actors, Transformation, Worldview, Ownership, Environmental constraints/aids).**

| **DOMAIN** | **Questions** | **Prompts applied to all placements models** |
| --- | --- | --- |
| **CATWOE MODEL** |  |  |
| **Customer** | 1. Who do you think benefits directly or indirectly from your placement? | - Student   - Depth of relationship   - Depth of learning   - Ability to change project   - Ability to reflect   - Team work - Supervisor - Community - University - Other - Rural specific issues (Lismore only) |
| **Actors** | 1. Who has been involved in your placement and what was your role and their role? | - Student - Supervisor - Community - University - Other - Rural specific issues (Lismore only) |
| **Transformation** | 1. What changes are required to improve the placement? | - Models of supervision - Elements needed to improve the system (input) (eg time, resources) - Where would it work best? - Any rural specific issues (Lismore only) |
| **Worldview** | 1. Discuss your views on integrated placements in training future Dietitians? | - Any rural specific issues (Lismore only) |
| **Ownership** | 1. Who/what can influence the success of Integrated Placements? | - Any rural specific issues (Lismore only) |
| **Environmental constraints** | See questions 1-5, 10 to identify barriers | - Student - Supervisor - Community - University - Other - Any rural specific issues (Lismore only) |
| **WORK READINESS** |  |  |
| **Ability to change systems or practice to reduce health inequity.** | 1. Can you describe whether you have been able to change systems or practices during your placement? | - If yes, please explain (how) - If no, why not |
| **Transformational leadership** | 1. Can you describe whether you have been able to empower other people during your placement? | - If yes, please explain (how) - If no, why not? - Staff/community / other students/ stakeholders |
| **Innovation ability** | 1. Can you describe whether you had opportunities to implement or adopt new ideas, processes, products or services? | - If yes, please explain (what did you do) - If no, why not? |
| **Ability to work inter-professionally and multi-disciplinary** | 1. Did your placement or placements, provide any multi-disciplinary and inter-professional education or training? How did this influence your learning? | - If yes, explain how this influenced your learning |

# **Supervisor questions**

1. Were the students aware of the type of placement they were on, and what it entails?
2. What benefits or challenges can you identity with the different styles of placement? For students and for supervisors?
3. How do the different styles of placement influence a student’s progression towards entry-level competency?
4. Do you feel that different placement styles suits students with specific learning styles or attributes?
5. Do you feel that different placement styles suit sites with specific features i.e., the size of the hospital or workloads of staff etc?
6. Is there anything about either type of placement style that you would change?
7. Do you have any feedback for the university based on your experiences?
8. Can you describe your experience of the integrated placement and how you managed as a supervisor?
